# Supplementary material for: The Sex Difference in 6-h Ultra-Marathon Running—The Worldwide Trends from 1982 to 2020
Source: Medicina (Kaunas). 2022 Jan 25;58(2):179. doi: 10.3390/medicina58020179 (PMC8876730; doi:10.3390/medicina58020179)
Supplement: Supplementary file 1 [file medicina-58-00179-s001.zip › medicina-1551617-supplementary.pdf]

SUPPLEMENTARY TABLES

Supplementary Table 1. Descriptive statistics (mean and standard deviation) and linear regression results to verify the predictors associated with running speed in female ultra-marathoners from different countries.

	**β**	***p*-value**	**Frequency (%)**	**Age (years)**	**Distance (km)**	**Running speed (km/h)**
				Mean (Std)	Mean (Std)	Mean (Std)
Russia	Reference	Reference	452 (1.95%)	38.89 (6.19)	60.06 (7.71)	10.01 (1.28)
Cyprus	-0.05736	0.93958	2 (0.01%)	34 (2.83)	57.11 (6.13)	9.93 (0.68)
Ukraine	-0.39729	0.02272*	42 (0.18%)	40.32 (11.16)	56.71 (7.66)	9.86 (2.14)
Island	-0.32848	0.31361	11 (0.05%)	33.18 (5.53)	58.35 (3.86)	9.72 (0.64)
Belgium	-0.1956	0.01131*	339 (1.46%)	44.58 (7.5)	58.2 (7.45)	9.7(1.24)
Monaco	-0.05927	0.95578	1 (0%)	41	58.15	9.69
Albania	-0.09429	0.81674	7 (0.03%)	43.86 (1.07)	57.78 (6.17)	9.63 (1.03)
Denmark	-0.1359	0.05213	615 (2.65%)	43.11 (8.17)	53.69 (6.31)	9.58 (1.16)
Poland	-0.16411	0.20192	98 (0.42%)	38.15 (7.71)	57.27 (8.43)	9.55 (1.4)
Croatia	-0.24846	0.16918	43 (0.19%)	35.13 (7.78)	50.11 (4.49)	9.52 (1.02)
Netherlands	-0.38433	<0.001*	545 (2.35%)	45.86 (7.47)	57.04 (6.84)	9.51 (1.14)
Romania	-0.39856	0.00868	56 (0.24%)	43.1 (9.52)	56.92 (6.78)	9.49 (1.13)
Hungary	-0.45297	<0.001*	826 (3.56%)	39.03 (7.41)	56.55 (6.86)	9.42 (1.14)
Norway	-0.3659	<0.001*	469 (2.02%)	44.66 (10.18)	56.25 (7.24)	9.38 (1.21)
Dominican Republic	-0.42139	0.69341	1 (0%)	49	57.45 (6.95)	9.35
New Zealand	-0.6458	0.00001*	63 (0.27%)	41.14 (8.81)	56.01 (6.46)	9.34 (1.08)
Portugal	0	<0.001*	5 (0.02%)	36.79 (9.5)	55.92 (4.89)	9.32 (0.82)
Austria	-0.60366	<0.001*	781 (3.37%)	43.14 (8.89)	55.83 (6.84)	9.3 (1.14)
Latvia	-0.66716	0.00631*	21 (0.09%)	37.3 (8.8)	55.77 (4.65)	9.3 (0.78)
Northern Macedonia	-0.78261	0.30115	2 (0.01%)	27 (2.83)	55.77 (10.98)	9.29 (1.83)
Spain	-0.53646	0.00001*	110 (0.47%)	40.19 (8.61)	49.9	9.28 (1.35)
Lithuania	-0.22253	0.47656	17 (0.07%)	37.5 (2.43)	55.57 (7.78)	9.26 (1.3)

South Korea	-0.52147	0.23473	9 (0.04%)	47.67 (4.18)	55.4 (4.76)	9.23 (0.79)
Finland	-0.61279	<0.001*	441 (1.9%)	42.49 (8.77)	54.44 (5.45)	9.21 (1.02)
Slovenia	-0.71067	<0.001*	170 (0.73%)	48 (7.24)	55.22 (7.52)	9.2 (1.25)
Thailand	-1.74941	0.00111*	4 (0.02%)	44.75 (4.57)	46.73	9.16 (1.11)
Japan	-0.50082	<0.001*	529 (2.28%)	45.15 (8.16)	54.71 (6.74)	9.12 (1.12)
Estonia	-0.80036	0.00009*	30 (0.13%)	39.14 (9.55)	55.66 (8.1)	9.07 (0.91)
Germany	-0.7584	<0.001*	4358 (18.8%)	45.46 (9.11)	52.32 (6.62)	9.07 (1.16)
Hong Kong	-0.74146	0.00002*	49 (0.21%)	42.4 (9.22)	54.37 (5.67)	9.06 (0.95)
France	-0.75715	<0.001*	2013 (8.68%)	46.74 (8.36)	55.23 (6.14)	9.04 (1.02)
China	-0.713	<0.001*	134 (0.58%)	38.12 (8.46)	53.44 (6.23)	9.03 (1.04)
Australia	-0.82947	<0.001*	658 (2.84%)	42.22 (10.46)	54.14 (6.57)	9.02 (1.1)
Ireland	-0.46293	0.0164*	48 (0.21%)	40.3 (5.39)	54.11 (6.89)	9.02 (1.15)
Italy	-0.76297	<0.001*	2662 (11.48%)	44.72 (8.45)	54.09 (6.65)	9.02 (1.11)
Madagascar	-0.49379	0.01443*	33 (0.14%)	50.83 (6.42)	49.36 (5.85)	9.02 (0.77)
Argentina	-0.81259	<0.001*	169 (0.73%)	42.35 (8.53)	53.84 (6.03)	8.97 (1.01)
Slovakia	-1.10815	<0.001*	49 (0.21%)	36.66 (11.65)	53.84 (6.58)	8.97 (1.1)
Czech	-1.07824	<0.001*	224 (0.97%)	35.43 (8.68)	59.59 (4.07)	8.95 (1.05)
Canada	-1.01598	<0.001*	626 (2.7%)	41.89 (9.6)	46.35	8.93 (1.02)
Chile	-1.10336	0.02159*	5 (0.02%)	33.2 (2.17)	53.6 (6.13)	8.91 (1.04)
Sweden	-0.68258	<0.001*	1494 (6.44%)	41.01 (10.09)	53.42 (4.89)	8.9 (0.82)
Bosnia	-1.13915	0.01769*	5 (0.02%)	33 (6.28)	53.36 (6.33)	8.89 (1.05)
Bulgaria	-0.98704	<0.001*	41 (0.18%)	38.45 (8.56)	53.28 (5.41)	8.88 (0.9)
Greece	-0.98391	<0.001*	37 (0.16%)	41.66 (5.66)	53.22 (5.07)	8.87 (0.84)
Mongolia	-0.92415	0.01018*	11 (0.05%)	37.78 (12.62)	53.21 (6.34)	8.87 (1.06)
Switzerland	-0.91954	<0.001*	71 (0.31%)	44.76 (8.94)	53.16 (6.55)	8.86 (1.09)
Belarus	-1.12883	0.00006*	15 (0.06%)	43.8 (17.15)	53.02 (6.69)	8.84 (1.11)
Brazil	-1.07312	<0.001*	131 (0.56%)	41.46 (9.75)	52.66 (5.4)	8.78 (0.9)
Turkey	0.82468	0.27577	3 (0.01%)	44.5 (0.71)	59.14 (12.87)	8.77 (0.92)
Mexico	-1.13418	0.00291*	8 (0.03%)	37.25 (5.6)	54.14 (4.6)	8.75 (1.11)
Montenegro	-1.5308	0.00433*	4 (0.02%)	26.5 (14.48)	52.4 (2.2)	8.73 (0.37)
Great Britain	-0.83693	<0.001*	427 (1.84%)	44.45 (8.45)	54.26 (6.11)	8.72 (1.1)

Luxemburg	-0.82892	0.27334	3 (0.01%)	48.5 (3.54)	49.66 (3.13)	8.69 (0.55)
Paraguay	-1.37244	0.00032*	9 (0.04%)	34.13 (7.68)	51.66 (3.23)	8.61 (0.54)
South Africa	-1.22955	<0.001*	22 (0.09%)	39.79 (11.89)	51.39 (5.18)	8.56 (0.86)
Serbia	-1.07577	<0.001*	96 (0.41%)	39.23 (12.24)	51.37 (4.53)	8.56 (0.76)
Kazakhstan	-1.27672	0.00362*	6 (0.03%)	45.67 (14.31)	51.23 (5.48)	8.54 (0.91)
Colombia	-0.74786	0.48416	2 (0.01%)	48	54.18 (6.25)	8.35 (0.75)
Ecuador	-1.51436	0.15654	1 (0%)	44	56.13	8.32
Malaysia	-1.34487	0.00019*	16 (0.07%)	33 (7.14)	52.14	8.23 (0.97)
India	-1.61374	<0.001*	18 (0.08%)	38 (9.12)	49.33	8.22 (0.66)
Peru	-1.45911	0.00013*	8 (0.03%)	55.25 (7.48)	48.53	8.09 (0.39)
Taiwan	-0.95756	<0.001*	538 (2.32%)	43.56 (8.25)	54.99	7.99 (0.27)
Philippines	-1.75254	<0.001*	13 (0.06%)	48.45 (6.79)	47.6	7.93 (0.31)
Nigeria	-2.01877	0.05893	1 (0%)	39	47.48	7.91
Sint Maarten	-0.82579	0.43978	1 (0%)	40.64 (9.18)	53.96	7.84 (1.17)
Iran	-2.0425	0.05602	1 (0%)	48	46.38	7.73
Uruguay	-0.74809	<0.001*	88 (0.38%)	70.4 (53.71)	12.28	
United States	-1.27611	<0.001*	3371 (14.54%)	70 (40.69)	15.21	
Venezuela	-0.4645	0.66389	1 (0%)	55.88 (55.88)	9.31	
Constant	52.8911	<0.001*				
Age	0.10452	0.71356				
Year event	-0.02093	0.00071*				
Year x Age	-0.00006	0.66075				

Note: * indicates statistically significant differences for running speed, compared to Russia. Results are presented as mean and standard deviation.

Supplementary Table 2. Descriptive statistics (mean and standard deviation) and linear regression results to verify the predictors associated with running speed in male ultra-marathoners from different countries.

	β	*p*-value	Frequency (%)	Age (years)	Distance (km)	Running speed (km/h)
				Mean (Std)	Mean (Std))	Mean (Std)
Tunisia	Reference	Reference	17 (0.0%)	44.82 (4.59)	72.95 (8.02)	12.16 (1.46)
Sri Lanka	-0.023	0.986	1 (0.0%)	36.00	71.42 (3.95)	11.90 (11.90)
Malta	0.177	0.804	4 (0.0%)	61.00 (0.82)	69.19(10.39)	11.53 (0.75)
Cape Verde	-0.708	0.181	9 (0.0%)	35.22 (4.29)	67.82(7.71)	11.30 (1.46)
Montenegro	-1.127	0.240	2 (0.0%)	40.50 (7.78)	65.70(8.31)	10.95 (2.29)
Madagascar	-1.452	0.006*	11 (0.0%)	37.44 (7.00)	65.01(8.40)	10.83 (1.64)
Macau	-1.169	0.376	1 (0.0%)	-	64.88	10.81
Belgium	-1.374	<0.001*	2784 (3.2%)	45.61 (9.59)	64.13(9.84)	10.69 (1.64)
France	-2.225	<0.001*	10932 (12.5%)	46.68 (9.64)	57.79(9.34)	9.63 (1.32)
Faroe Islands	-0.919	0.159	5 (0.0%)	52.80 (2.59)	63.63(8.85)	10.61 (1.37)
Russia	-1.467	<0.001*	2043 (2.3%)	43.68 (11.94)	63.49	10.58 (1.60)
Botswana	-2.434	0.065	1 (0.0%)	35.00	63.20(7.44)	10.53
Venezuela	-1.337	0.096	3 (0.0%)	41.00 (4.36)	62.51	10.42 (0.33)
Lithuania	-1.257	<0.001*	123 (0.1%)	42.08 (8.26)	62.48(6.55)	10.41 (1.39)
Andorra	-1.195	0.094	4 (0.0%)	50.75 (11.35)	62.34(6.97)	10.39 (1.73)
Latvia	-1.591	<0.001*	61 (0.1%)	42.55 (8.93)	61.24(7.11)	10.21 (1.42)
Netherlands	-1.899	<0.001*	3479 (4.0%)	48.41 (9.25)	60.32(7.90)	10.05 (1.34)
Hungary	-1.877	<0.001*	1969 (2.3%)	41.25 (8.57)	60.04	10.01 (1.37)
Honduras	-1.825	0.167	1 (0.0%)	46.00	60 (8.75)	10.00
Iceland	-2.07	<0.001*	15 (0.0%)	44.54 (8.01)	59.56(7.22)	9.93 (0.88)
Spain	-1.867	<0.001*	1185 (1.4%)	43.91 (9.29)	59.43(4.28)	9.91 (1.55)
Denmark	-1.954	<0.001*	2089 (2.4%)	42.92 (8.02)	59.41(7.78)	9.90 (1.23)
Morocco	-1.422	0.046*	5 (0.0%)	45.00 (6.83)	59.39(7.40)	9.90 (2.01)
Belarus	-2.139	<0.001*	151 (0.2%)	48.30 (13.36)	59.18 (5.91)	9.86 (1.47)
Bosnia	-2.043	<0.001*	40 (0.0%)	40.88 (10.43)	58.90	9.82 (1.56)
Norway	-1.974	<0.001*	1465 (1.7%)	45.88 (11.09)	58.87 (9.30)	9.81 (1.46)

Austria	-2.133	<0.001*	3302 (3.8%)	43.37 (9.82)	58.84 (7.74)	9.81 (1.40)
Slovenia	-2.139	<0.001*	506 (0.6%)	41.69 (8.92)	58.78 (7.62)	9.80 (1.47)
Peru	-1.772	<0.001*	25 (0.0%)	34.39 (3.26)	58.62 (7.95)	9.77 (1.52)
Romania	-1.963	<0.001*	114 (0.1%)	39.06 (8.44)	58.60 (8.23)	9.77 (1.36)
Switzerland	-2.057	<0.001*	320 (0.4%)	45.79 (10.18)	58.55 (8.31)	13.26 (9.76)
Ukraine	-2.216	<0.001*	142 (0.2%)	40.91 (11.05)	58.52 (4.90)	9.75 (1.38)
Poland	-2.06	<0.001*	421 (0.5%)	38.09 (8.56)	58.38 (8.19)	9.73 (1.72)
Slovakia	-2.306	<0.001*	358 (0.4%)	41.27 (11.30)	58.33 (7.49)	14.07 (9.72)
Czechia	-2.33	<0.001*	584 (0.7%)	39.88 (10.36)	58.30 (2.85)	9.72 (1.30)
Greenland	-2.093	0.001*	5 (0.0%)	45.80 (4.87)	57.98 (7.18)	9.66 (0.47)
Mongolia	-2.512	<0.001*	22 (0.0%)	30.22 (7.67)	57.86	9.64 (1.14)
Germany	-2.189	<0.001*	14452 (16.6%)	47.78 (9.80)	57.70 (8.21)	9.62 (1.36)
Estonia	-2.254	<0.001*	68 (0.1%)	41.74 (9.26)	57.69	9.62 (1.29)
Kazakhstan	-2.368	<0.001*	45 (0.1%)	40.03 (13.06)	57.62 (8.04)	9.60 (1.35)
Australia	-2.363	<0.001*	1709 (2.0%)	44.15 (9.91)	57.48	9.58 (1.38)
Sweden	-2.229	<0.001*	3884 (4.5%)	45.06 (10.54)	57.44 (0.96)	15.26 (9.57)
Argentine	-2.196	<0.001*	429 (0.5%)	43.24 (9.35)	57.31 (8.96)	9.55 (1.29)
Finland	-2.25	<0.001*	937 (1.1%)	45.18 (9.86)	57.31 (5.29)	9.55 (1.27)
Japan	-2.122	<0.001*	2048 (2.3%)	44.07 (9.92)	57.05 (7.70)	9.51 (1.42)
South Africa	-2.357	<0.001*	88 (0.1%)	40.56 (8.45)	56.99	9.50 (1.56)
Serbia	-2.309	<0.001*	335 (0.4%)	45.02 (13.18)	56.91 (8.55)	9.48 (1.29)
New Zealand	-2.482	<0.001*	141 (0.2%)	44.29 (11.01)	56.86 (8.09)	9.48 (1.16)
Portugal	-2.268	<0.001*	88 (0.1%)	41.67 (9.44)	56.80 (6.63)	9.47 (1.33)
Ireland	-2.064	<0.001*	102 (0.1%)	42.62 (9.32)	56.72 (8.51)	9.45 (1.49)
Croatia	-2.358	<0.001*	92 (0.1%)	42.08 (9.58)	56.68 (8.33)	9.45 (1.20)
Ecuador	-2.748	<0.001*	11 (0.0%)	32.18 (7.99)	56.63 (7.68)	9.44 (0.98)
China	-2.267	<0.001*	518 (0.6%)	40.33 (10.42)	56.53	9.42 (1.32)
Luxembourg	-2.577	<0.001*	41 (0.0%)	43.49 (11.72)	56.50 (12.03)	9.42 (1.28)
Greece	-2.524	<0.001*	342 (0.4%)	42.33 (8.91)	56.48 (4.23)	9.41 (1.25)
Bulgaria	-2.525	<0.001*	137 (0.2%)	36.68 (8.49)	56.25 (9.86)	9.38 (1.09)
Uruguay	-2.399	<0.001*	196 (0.2%)	41.96 (9.15)	56.24 (6.91)	9.37 (1.12)

Albania	-2.976	<0.001*	5 (0.0%)	30.60 (7.13)	56.16 (6.86)	9.36 (1.34)
Singapura	-2.542	<0.001*	9 (0.0%)	39.38 (3.66)	56.08 (6.75)	9.35 (1.10)
Brazil	-2.446	<0.001*	550 (0.6%)	41.40 (9.04)	56.00 (4.52)	9.33 (1.24)
San Marino	-1.925	<0.001*	13 (0.0%)	59.77 (6.43)	55.99 (13.72)	9.33 (0.89)
Cyprus	-2.85	<0.001*	6 (0.0%)	32.33 (8.24)	55.95 (3.86)	9.33 (0.71)
Italy	-2.337	<0.001*	13633 (15.6%)	49.31 (9.43)	55.89 (8.06)	9.32 (1.28)
Jordan	-2.253	0.088	1 (0.0%)	49.00	55.78 (2.67)	9.30
South Korea	-2.656	<0.001*	78 (0.1%)	46.09 (8.16)	55.71 (8.74)	9.28 (1.11)
Azerbaijan	-2.52	0.056	1 (0.0%)	39.00	55.69 (6.98)	9.28
Puerto Rico	-2.422	0.067	1 (0.0%)	45.00	55.69	9.28
Great-Britain	-2.345	<0.001*	1252 (1.4%)	45.44 (9.29)	55.53	9.25 (1.38)
Algeria	-2.692	<0.001*	11 (0.0%)	45.64 (11.23)	55.52 (6.77)	9.25 (0.66)
Canada	-2.781	<0.001*	1586 (1.8%)	47.27 (11.28)	55.18 (9.10)	9.20 (1.16)
India	-2.386	<0.001*	122 (0.1%)	36.32 (9.69)	55.17 (5.54)	9.20 (1.34)
Hong Kong	-2.45	<0.001*	202 (0.2%)	45.52 (11.04)	54.69 (10.32)	9.11 (1.20)
Northern Macedonia	-2.766	<0.001*	11 (0.0%)	41.73 (10.20)	54.69 (7.97)	9.11 (1.12)
USA	-2.893	<0.001*	6949 (8.0%)	43.61(10.95)	53.93	8.99 (1.16)
Taiwan	-2.754	<0.001*	5 (0.0%)	46.74 (9.34)	53.49 (8.16)	8.91 (1.04)
Mexico	-2.83	<0.001*	56 (0.1%)	39.05 (9.01)	53.37 (9.34)	8.89 (1.15)
Turkey	-2.937	<0.001*	39 (0.0%)	41.57 (8.88)	53.08 (9.61)	8.85 (1.04)
Philippines	-2.934	<0.001*	28 (0.0%)	41.67 (10.32)	52.73 (4.44)	8.79 (0.92)
Paraguay	-2.938	<0.001*	14 (0.0%)	36.17 (6.55)	52.64 (6.61)	8.77 (1.13)
Iran	-3.091	0.001*	2 (0.0%)	46.00 (14.14)	52.63 (8.82)	8.77 (0.16)
Chile	-3.24	<0.001*	40 (0.0%)	32.45 (3.36)	52.38 (5.34)	8.73 (1.19)
Nicaragua	-3.094	0.001*	2 (0.0%)	44.00	51.15 (7.75)	8.53 (0.64)
Indonesia	-3.059	0.021*	1 (0.0%)	51.00	50.60	8.43
Malaysia	-3.222	<0.001*	60 (0.1%)	41.03 (10.94)	50.59 (8.99)	8.43 (0.71)
Nigeria	-3.864	<0.001*	4 (0.0%)	28.25 (1.89)	50.28 (7.57)	8.38 (0.44)
Georgia	-4.165	0.002*	5 (0.0%)	38.00	50.08 (7.94)	8.35 (0.82)
Brunei	-3.613	0.006*	1 (0.0%)	34.00	50.00 (0.57)	8.33
Senegal	-3.383	<0.001*	3 (0.0%)	46.67 (3.06)	49.94 (6.25)	8.32 (0.74)

Panama	-3.426	0.009*	1 (0.0%)	45.00	49.89 (8.78)	8.32
Columbia	-3.198	0.015*	1 (0.0%)	51.00	49.54 (6.23)	8.26
Eritrea	-4.191	0.001*	1 (0.0%)	26.00	48.59 (8.25)	8.10
Tanzania	-3.033	0.002*	2	65.00	47.88 (6.99)	8.05 (7.98)
IRE	-3.782	0.004*	1 (0.0%)	52.00	46.09 (2.00)	7.68
Oman	-4.78	<0.001*	1 (0.0%)	24.00	45.40 (2.48)	7.57
Constant	87.607	<0.001				
Event year	-0.037	<0.001*				
Age x year	-1.43	<0.001*				

Note: * indicates statistically significant differences for running speed, compared to Tunisia. Results are presented as mean and standard deviation.

Supplementary Table 3. Descriptive statistics (mean and standard deviation) and linear regression results to verify the predictors associated with running speed in the top 10 female ultra-marathoners from different countries.

	**β**	***p*-value**	**Age (years)**	**Distance (km)**	**Running speed (km/h)**
			Mean (Std)	Mean (Std)	Mean (Std)
Slovenia	Reference	Reference	39.06 (6.06)	68.28 (6.33)	11.38 (1.06)
Norway	0.25	<0.001*	41.23 (10.25)	66.24 (6.17)	11.04 (1.03)
Poland	0.54	<0.001*	37.83 (7.25)	65.55 (9.16)	10.92 (1.53)
Spain	-0.06	0.62	38.15 (8.08)	64.90 (5.97)	10.82 (0.99)
Belgium	0.57	<0.001*	42.02(5.99)	64.55 (6.93)	10.76 (1.16)
Russia	0.80	<0.001*	36.57 (10.84)	63.75 (8.41)	10.62 (1.40)
Sweden	0.09	0.10	37.67 (8.57)	63.63 (6.59)	10.61 (1.10)
Hungry	0.21	<0.001*	38.27 (6.41)	63.52 (6.45)	10.59 (1.08)
Netherlands	0.28	<0.001*	45.36 (7.23)	63.29 (6.39)	10.55 (1.06)
Austria	0.19	<0.001*	41.35 (8.69)	62.74 (6.74)	10.46 (1.12)
Romania	0.09	0.58	35.67 (8.73)	62.69 (5.72)	10.45 (0.95)
Denmark	0.56	<0.001*	42.10 (7.02)	62.63 (7.50)	10.44 (1.25)
Italy	-0.03	0.58	41.48 (7.26)	62.16 (7.04)	10.36 (1.17)
Finland	0.09	0.19	39.88 (9.30)	60.88 (5.85)	10.15 (0.97)
Japan	0.18	0.01*	44.03 (6.57)	60.67 (7.88)	10.11 (1.31)
Germany	0.06	0.22	43.00 (7.89)	60.47 (7.85)	10.08 (1.31)
Uruguay	-0.06	0.68	38.50 (6.86)	59.36 (4.57)	9.89 (0.76)
Czech	-0.25	<0.001*	34.31 (9.41)	58.99 (5.90)	9.83 (0.98)
France	-0.05	0.36	45.48 (7.45)	58.98 (6.59)	9.83 (1.10)
China	0.04	0.69	38.52 (9.40)	58.87 (7.48)	9.81 (1.25)
Croatia	0.61	<0.001*	33.42 (7.23)	58.87 (6.43)	9.81 (1.07)
Taipei	-0.09	0.16	42.64 (6.80)	58.76 (4.89)	9.79 (0.82)
Argentina	-0.22	0.03*	42.76 (8.79)	58.06 (6.48)	9.68 (1.08)
Slovakia	-0.45	0.01*	43.07 (13.06)	57.48 (3.36)	9.58 (0.56)
Ireland	-0.03	0.90	40.33 (3.54)	57.29 (7.00)	9.55 (1.17)
Serbia	-0.40	<0.001*	36.81 (11.53)	57.13 (7.98)	9.52 (1.33)
Canada	-0.49	<0.001*	39.91 (8.04)	57.06 (6.58)	9.51 (1.10)

Australia	-0.30	<0.001*	41.97 (11.17)	56.91(6.78)	9.48 (1.13)
Switzerland	-0.26	0.06	43.80 (9.23)	56.59 (7.25)	9.43 (1.21)
New Zealand	0.22	0.34	41.52 (9.37)	56.28(6.70)	9.38 (1.12)
Ukraine	0.68	<0.001*	33.38 (9.09)	55.51 (6.34)	9.25 (1.06)
Great Britain	-0.17	0.04*	45.01 (8.88)	54.11 (7.15)	9.02 (1.19)
USA	-0.65	<0.001*	39.84 (8.74)	53.49 (6.09)	8.91(1.02)
Brazil	-0.20	0.14	41.12 (9.99)	53.40 (5.69)	8.90 (0.95)
South Africa	-0.15	0.62	39.36 (7.37)	51.65 (6.47)	8.61 (1.08)
Malaysia	-0.26	0.69		49.97(6.20)	8.33 (1.03)
Constant	70.24	<0.001*			
Event year	-0.03	<0.001*			
Age x Year	0.00	<0.001*			

Note: * indicates statistically significant differences for running speed, compared to Slovenia. Results are presented as mean and standard deviation.

Supplementary Table 4. Descriptive statistics (mean and standard deviation) and linear regression results to verify the predictors associated with running speed in the top 100 female ultra-marathoners from different countries.

	β	*p*-value	Age (years)	Distance (km)	Running speed (km/h)
			Mean (Std)	Mean (Std)	Mean (Std)
Russia	Reference	Reference	42.27 (8.56)	53.82 (6.06)	8.97 (1.01)
Belgium	-0.034	0.644	42.30 (10.41)	54.08 (6.59)	9.01 (1.10)
Iceland	-0.171	0.598	43.19 (8.77)	56.04 (6.84)	9.34 (1.14)
Ukraine	-0.235	0.175	44.45 (7.45)	58.52 (7.50)	9.75 (1.25)
Denmark	0.026	0.694	41.37 (9.66)	52.58 (5.46)	8.76 (0.91)
Poland	-0.003	0.984	39.03 (7.89)	53.28 (5.41)	8.88 (0.90)
Netherland	-0.219	0.001	42.03 (9.51)	53.58 (6.15)	8.93 (1.02)
Croatia	-0.089	0.620	38.59 (8.20)	54.85 (6.30)	9.14 (1.05)
Romania	-0.237	0.116	34.25 (8.00)	57.31 (6.62)	9.55 (1.10)
Norway	-0.197	0.005*	35.27 (8.82)	53.68 (6.45)	8.95 (1.07)
Hungry	-0.290	<0.001*	42.97 (8.12)	57.94 (6.88)	9.66 (1.15)
Spain	-0.377	0.002*	40.00 (9.50)	54.81 (5.74)	9.14 (0.96)
Austria	-0.440	<0.001*	42.40 (8.85)	55.26 (6.21)	9.21 (1.04)
New Zealand	-0.487	0.001*	46.77 (8.30)	54.28 (6.11)	9.05 (1.02)
Sweden	-0.516	<0.001*	45.03 (8.80)	55.44 (7.37)	9.24 (1.23)
Latvia	-0.505	0.038*	44.65 (8.57)	52.21 (6.66)	8.70 (1.11)
Germany	-0.595	<0.001*	40.79 (5.63)	53.44 (4.91)	8.91 (0.82)
Slovenia	-0.551	<0.001*	42.40 (9.22)	54.37 (5.67)	9.06 (0.95)
Lithuania	-0.059	0.849	39.07 (7.34)	56.56 (6.89)	9.43 (1.15)
Finland	-0.450	<0.001*	40.00 (9.53)	8.17 (0.69)	49.02 (4.16)
Madagascar	-0.325	0.106	40.48 (5.60)	54.04 (7.34)	9.01 (1.22)
China	-0.551	<0.001*	33.18 (5.53)	58.35 (3.86)	9.72 (0.64)
Japan	-0.337	<0.001*	44.47 (8.42)	54.62 (6.67)	9.10 (1.11)
Estonia	-0.641	0.002*	45.18 (8.07)	54.81 (6.75)	9.14 (1.13)
Italy	-0.599	<0.001*	36.67 (9.67)	55.69 (5.24)	9.28 (0.87)
Switzerland	-0.758	<0.001*	37.80 (2.15)	9.22 (1.34)	55.34 (8.03)
Hong Kong	-0.577	0.001*	50.75 (6.64)	54.96 (4.23)	9.16 (0.71)

France	-0.592	<0.001*	33.00 (7.14)	8.23 (0.97)	49.36 (5.85)
Australia	-0.663	<0.001*	37.78 (12.62)	8.87 (1.06)	53.21 (6.34)
Ireland	-0.300	0.118	45.90 (7.25)	57.63 (6.83)	9.60 (1.14)
Argentina	-0.649	<0.001*	40.65 (8.69)	55.97 (6.52)	9.33 (1.09)
Uruguay	-0.585	<0.001*	44.46 (9.52)	56.95 (7.03)	9.49 (1.17)
Czech	-0.926	<0.001*	48.45 (6.79)	47.60 (1.84)	7.93 (0.31)
Slovakia	-0.920	<0.001*	38.04 (7.96)	57.66 (8.69)	9.61 (1.45)
Canada	-0.850	<0.001*	36.82 (9.41)	57.23 (6.84)	9.54 (1.14)
Greece	-0.822	<0.001*	39.98 (11.64)	60.23 (7.66)	10.04 (1.28)
Bulgaria	-0.820	<0.001*	47.75 (7.28)	51.53 (4.56)	8.59 (0.76)
Mongolia	-0.762	0.034*	40.09 (11.50)	53.13 (6.59)	8.86 (1.10)
Taipei	-0.793	<0.001*	37.51 (11.50)	53.66 (4.83)	8.94 (0.80)
Serbia	-0.890	<0.001*	40.93 (9.08)	55.41 (7.51)	9.24 (1.25)
Brazil	-0.911	<0.001*	43.83 (9.70)	51.87 (5.55)	8.64 (0.92)
Great Britain	-0.673	<0.001*	39.98 (8.60)	56.13 (8.20)	9.35 (1.37)
South Africa	-1.066	<0.001*	40.96 (10.14)	55.91 (6.61)	9.32 (1.10)
USA	-1.113	<0.001*	45.13 (9.07)	54.40 (6.64)	9.07 (1.11)
San Marino	-0.937	<0.001*	43.29 (7.83)	53.21 (5.58)	8.87 (0.93)
Malaysia	-1.184	<0.001*	39.58 (9.99)	58.08 (8.10)	9.68 (1.35)
Indonesia	-1.450	<0.001*	40.73 (9.32)	51.65 (5.62)	8.61 (0.94)
Philippines	-1.585	<0.001*	41.70 (7.91)	53.69 (5.85)	8.95 (0.98)
Constant	58.520	<0.001*			
Event year	-0.024	<0.001*			
Age x Year	-1.051	<0.001*			

Note: * indicates statistically significant differences for running speed compared to Russia. Results are presented as mean and standard deviation.

Supplementary Table 5. Descriptive statistics (mean and standard deviation) and linear regression results to verify the predictors associated with running speed in the top 10 male ultra-marathoners from different countries.

	**β**	***p*-value**	**Age (years)**	**Distance (km)**	**Running speed (km/h)**
			Mean (Std)	Mean (Std)	Mean (Std)
Tunisia	Reference	Reference	45.47 (3.34)	75.52 (4.64)	12.59 (0.77)
Belgium	1.97	<0.001*	43.59 (8.10)	73.50 (7.73)	12.25 (1.29)
Russia	1.54	<0.001*	40.10 (11.08)	70.84 (9.06)	15.54 (11.81)
Norway	1.67	<0.001*	43.01 (9.88)	70.11 (7.19)	14.80 (11.69)
Slovenia	1.38	<0.001*	39.05 (8.73)	69.11 (7.17)	11.52 (1.19)
Lithuania	1.51	<0.001*	40.59 (7.25)	68.71 (6.71)	14.88 (11.45)
Netherland	1.10	<0.001*	46.24 (8.40)	62.35 (5.82)	14.54 (11.19)
Poland	1.20	<0.001*	37.98 (9.12)	67.10 (10.09)	15.37 (11.18)
Spain	1.13	<0.001*	42.23 (8.94)	66.80 (8.15)	11.13 (1.36)
Hungary	0.97	<0.001*	39.91 (7.83)	66.26 (7.54)	11.04 (1.26)
Denmark	1.05	<0.001*	40.86 (7.56)	66.23 (7.90)	11.04 (1.32)
Sweden	0.95	<0.001*	41.36 (8.93)	66.01 (7.79)	11.00 (1.30)
Latvia	0.98	<0.001*	42.76 (7.95)	65.20 (7.50)	11.45 (1.12)
Germany	0.83	<0.001*	45.12 (9.01)	64.99 (8.37)	10.83 (1.39)
Italy	0.89	<0.001*	45.07 (8.25)	64.81 (7.75)	10.65 (1.47)
Finland	0.80	<0.001*	44.03 (9.16)	64.54 (6.89)	10.76 (1.15)
Romania	0.70	<0.001*	37.68 (8.43)	64.16 (7.13)	13.13 (10.69)
Switzerland	0.78	<0.001*	45.99 (9.28)	64.12 (8.04)	10.69 (1.34)
France	0.62	<0.001*	45.17 (8.86)	63.99 (7.63)	10.67 (1.27)
China	0.74	<0.001*	37.21 (9.99)	63.99 (8.44)	10.66 (1.41)
Japan	0.67	<0.001*	42.95 (9.81)	63.87 (8.84)	9.37 (1.25)
Czech	0.30	0.001*	37.79 (9.38)	63.50 (6.73)	10.58 (1.12)
Bosnia	0.48	0.069	39.96 (9.97)	63.09 (9.28)	10.51 (1.55)
Slovakia	0.29	0.004*	39.92 (9.91)	63.08 (6.69)	10.51 (1.11)
Hong Kong	0.67	0.001*	42.10 (10.27)	62.95 (5.51)	10.49 (0.92)
Belarus	0.32	0.033*	46.51 (13.45)	62.92 (8.83)	10.49 (1.47)
Argentina	0.47	<0.001*	43.05 (8.49)	62.55 (6.74)	10.42 (1.12)

Greece	0.30	0.009*	42.03 (8.36)	62.22 (6.91)	10.37 (1.15)
Bulgaria	0.28	0.110	36.62 (8.26)	62.20 (4.54)	10.37 (0.76)
Uruguay	0.35	0.027*	40.94 (8.79)	61.54 (6.06)	10.26 (1.01)
Croatia	0.31	0.204	39.65 (9.14)	61.23 (6.25)	10.20 (1.04)
South Africa	0.16	0.436	41.25 (7.62)	61.21 (10.67)	13.87 (10.20)
Taipei	0.30	<0.001*	45.25 (8.52)	60.89 (6.19)	10.15 (1.03)
Estonia	0.07	0.730	41.27 (8.31)	60.59 (8.24)	10.10 (1.37)
Austria	1.16	<0.001*	40.78 (8.75)	67.79 (7.37)	11.30 (1.23)
Iceland	-0.02	0.955	47.89 (7.30)	60.54 (5.80)	10.80 (1.29)
Portugal	-0.08	0.752	38.41 (10.33)	60.22 (8.24)	12.28 (10.04)
Serbia	0.03	0.761	42.87 (11.44)	60.05 (7.68)	10.01 (1.28)
Ukraine	-0.19	0.193	37.35 (10.14)	59.58 (8.99)	9.93 (1.50)
Brazil	-0.12	0.195	41.12 (8.69)	58.81 (7.56)	9.80 (1.26)
Mongolia	-0.49	0.178	30.67 (7.99)	58.66 (7.37)	11.55 (9.78)
Ireland	0.09	0.673	43.76 (9.78)	58.17 (9.62)	10.09 (0.97)
Canada	-0.37	<0.001*	44.62 (10.42)	58.58 (7.37)	9.76 (1.23)
Korea	-0.38	0.090	45.72 (7.50)	58.16 (7.05)	10.87 (1.25)
New Zealand	-0.46	<0.001*	44.44 (11.60)	58.01 (7.15)	12.27 (9.67)
Great Britain	-0.08	0.236	45.16 (9.25)	57.60 (8.82)	9.60 (1.47)
Luxembourg	-0.47	0.144	39.00 (12.81)	57.47 (9.71)	12.64 (9.58)
United States	-0.66	<0.001*	41.40 (10.04)	56.36 (7.43)	9.39 (1.24)
Kazakhstan	-0.80	0.003*	43.09 (14.50)	56.21 (7.49)	9.69 (1.18)
Indonesia	-0.34	0.077	35.71 (11.26)	55.83 (8.91)	9.31 (1.49)
Mexico	-1.04	<0.001*	38.00 (7.00)	54.30 (8.22)	11.53 (9.05)
Philippines	-1.04	0.004*	40.67 (11.38)	52.74 (5.67)	10.83 (8.79)
Paraguay	-1.48	0.017*	35.00 (5.35)	51.70 (6.62)	10.67 (8.62)
Malaysia	-1.47	<0.001*	38.19 (8.38)	50.37 (4.16)	10.20 (8.39)
Constant	86.62	<0.001*			
Event year	-0.04	<0.001*			
Age x Year	0.00	<0.001*			

Note: * indicates statistically significant differences for running speed compared to Tunisia. Results are presented as mean and standard deviation.

Supplementary Table 6. Descriptive statistics (mean and standard deviation) and linear regression results to verify the predictors associated with running speed in the top 100 male ultra-marathoners from different countries.

	**β**	***p*-value**	**Age (years)**	**Distance (km)**	**Running speed (km/h)**
			Mean (Std)	Mean (Std)	Mean (Std)
Tunisia	Reference	Reference	44.82 (4.59)	72.95 (8.78)	12.16 (1.46)
Belgium	0.62	<0.001*	45.60 (9.53)	64.78 (9.63)	10.80 (1.60)
France	-0.32	<0.001*	46.68 (9.61)	57.85 (7.94)	9.64 (1.32)
Lithuania	0.67	<0.001*	41.43 (7.68)	62.76 (8.36)	0.46 (1.39)
Latvia	0.43	0.03*	42.86 (8.96)	61.99 (8.81)	10.331.47)
Hungary	0.02	0.81	41.27 (8.57)	60.11 (8.19)	10.02 (1.36)
Iceland	-0.10	0.80	45.33 (7.81)	59.93 (5.28)	9.99 (0.88)
Denmark	-0.02	0.85	42.86 (7.91)	59.72 (7.33)	9.95 (1.22)
Bosnia	-0.15	0.52	40.19 (10.52)	59.18 (9.76)	9.86 (1.63)
Austria	-0.15	0.08	43.67 (9.76)	59.41 (8.25)	9.90 (1.37)
Slovenia	-0.22	0.03*	41.76 (8.90)	59.05 (8.79)	9.84 (1.47)
Romania	-0.05	0.72	39.14 (8.55)	59.35 (8.00)	9.89 (1.33)
Switzerland	0.10	0.36	46.74 (9.60)	60.07 (8.52)	10.01 (1.42)
Poland	0.31	0.01*	38.62 (9.04)	61.07 (10.01)	10.18 (1.67)
Slovakia	-0.35	0.00*	41.52 (11.32)	58.74 (7.45)	9.79 (1.24)
Mongolia	-0.65	0.04*	30.22 (7.67)	57.86 (6.86)	9.64 (1.14)
Germany	-0.12	0.15	47.36 (9.67)	58.85 (8.28)	9.81 (1.38)
Kazakhstan	-0.48	0.04*	40.03 (13.06)	57.62 (8.09)	9.60 (1.35)
Australia	-0.46	<0.001*	44.17 (9.93)	57.57 (8.30)	9.60 (1.38)
Argentina	-0.30	0.00*	43.33 (9.37)	57.39 (7.71)	9.57 (1.28)
Finland	-0.36	<0.001*	45.20 (9.87)	57.28 (7.65)	9.55 (1.27)
South Africa	-0.39	0.02*	40.90 (8.45)	57.68 (9.41)	9.61 (1.57)
Serbia	-0.42	<0.001*	45.04 (13.18)	56.91 (7.79)	9.48 (1.30)
Portugal	-0.27	0.15	41.16 (9.55)	58.75 (7.81)	9.79 (1.30)
Ireland	-0.14	0.44	42.46 (9.39)	57.85 (9.09)	9.64 (1.52)
Ecuador	-0.87	0.03*	32.18 (7.99)	56.63 (5.91)	9.44 (0.98)

China	-0.28	0.01*	40.41 (10.40)	57.73 (7.71)	9.62 (1.28)
Greece	-0.63	<0.001*	42.37 (8.95)	56.49 (7.53)	9.41 (1.26)
Bulgaria	-0.65	<0.001*	36.68 (8.49)	56.25 (6.55)	9.38 (1.09)
Brazil	-0.51	<0.001*	41.57 (9.13)	56.36 (7.43)	9.39 (1.24)
San Marino	-0.02	0.96	59.77 (6.43)	55.99 (5.34)	9.330.89)
Korea	-0.76	<0.001*	46.09 (8.16)	55.71 (6.63)	9.28 (1.11)
Great-Britain	-0.41	<0.001*	45.51 (9.29)	55.77 (8.35)	9.29 (1.39)
Canada	-0.88	<0.001*	47.28 (11.29)	55.19 (6.98)	9.20 (1.16)
Indonesia	-0.49	0.00*	36.40 (9.66)	55.17 (8.18)	9.20 (1.36)
United States	-1.00	<0.001*	43.61 (10.95)	53.96 (6.99)	8.99 (1.16)
Taipei	-0.75	<0.001*	46.64 (9.19)	54.12 (6.33)	9.02 (1.06)
Turkey	-0.76	0.00*	42.68 (8.94)	54.67 (6.49)	9.11 (1.08)
Philippines	-1.05	0.00*	41.67 (10.32)	52.73 (5.54)	8.79 (0.92)
Chile	-0.18	0.78	32.00 (4.08)	59.82 (8.16)	9.97 (1.36)
Estonia	-0.34	0.07	41.50 (8.69)	57.92 (8.19)	9.65 (1.36)
Sweden	-0.23	0.01*	44.87 (10.41)	58.22 (7.81)	9.70 (1.30)
Japan	-0.19	0.03*	44.17 (9.93)	57.31 (8.52)	9.55 (1.42)
New Zealand	-0.59	<0.001*	44.37 (11.06)	57.00 (6.96)	9.50 (1.16)
Croatia	-0.21	0.22	42.45 (9.82)	58.30 (6.69)	9.72 (1.12)
Luxembourg	-0.27	0.31	41.96 (12.49)	58.95 (8.26)	9.82(1.38)
Uruguay	-0.51	<0.001*	42.09 (9.21)	56.23 (6.78)	9.37 (1.13)
Italy	-0.36	<0.001*	49.12 (9.37)	56.44(7.67)	9.41 (1.28)
Algeria	-0.80	0.04*	45.64 (11.23)	55.52 (3.95)	9.25 (0.66)
Hong Kong	-0.55	<0.001*	45.59 (11.04)	54.76 (7.17)	9.13 (1.20)
Madagascar	0.44	0.31	37.44 (7.00)	65.01 (9.86)	10.83 (1.64)
Mexico	-0.86	0.00*	39.70 (8.49)	55.03 (7.89)	9.17 (1.32)
Paraguay	-1.48	0.02*	35.00 (5.35)	51.70 (6.62)	8.62 (1.10)
Malaysia	-1.34	<0.001*	41.03 (10.94)	50.59 (4.23)	8.43 (0.71)
Russia	0.45	<0.001*	43.75 (11.93)	63.69 (9.51)	10.62 (1.59)
Netherland	0.13	0.13	48.37 (9.22)	61.02 (7.99)	10.17 (1.33)
Spain	0.04	0.64	43.95 (9.31)	59.66 (9.30)	9.94 (1.55)

Norway	0.00	0.99	46.04 (11.09)	59.33 (8.62)	9.89 (1.44)
Peru	0.62	0.08	34.29 (2.09)	62.80 (7.92)	10.47 (1.32)
Ukraine	-0.26	0.07	39.98 (10.86)	59.16 (8.44)	9.86 (1.41)
Czech	-0.44	<0.001*	39.80 (10.42)	58.37 (7.80)	9.73 (1.30)
Constant	83.26	<0.001*			
Event year	-0.04	<0.001*			
Age x year	0.00	<0.001*			

Note: * indicates statistically significant differences for running speed, compared to Tunisia. Results are presented as mean and standard deviation.
